# Potential Impact of Revising the Diagnostic Criteria for Gestational Diabetes on Maternal Pregnancy and Infant Perinatal Outcomes

**DOI:** 10.1111/1753-0407.70232

**Published:** 2026-05-12

**Authors:** Qiliang Liu, Jane E. Harding, Greg D. Gamble, Carl Eagleton, Lisa Dawes, Caroline A. Crowther

**Affiliations:** ^1^ Liggins Institute, University of Auckland, New Zealand Auckland New Zealand; ^2^ Te Whatu Ora Te Toka Tumai Auckland, Auckland, New Zealand Auckland New Zealand

**Keywords:** diabetes, diagnosis, gestational, glucose tolerance test, pregnancy outcome

## Abstract

**Aims:**

The revised diagnostic criteria for gestational diabetes (GDM) in New Zealand include lowering the fasting glucose from the current ≥ 5.5 to ≥ 5.3 mmol/L and replacing the current 2‐h post‐load glucose (≥ 9.0 mmol/L) with a 1‐h glucose (≥ 10.6 mmol/L). We assessed the effects of these changes on the demographics of those diagnosed and their perinatal outcomes.

**Materials and Methods:**

Participants in the GEMS trial were grouped as meeting only the revised criteria (Additional group), only the current criteria (Missed group), both criteria (Both‐Criteria group), and neither criteria (Non‐GDM group).

**Results:**

GDM prevalence among 3921 participants was 6.8% using the revised criteria and 5.8% using the current criteria (risk ratio 1.18, 95% CI 1.00–1.40). Women in the Additional (*n* = 125) and Missed groups (*n* = 83), who would be affected by the changes, were more likely to be overweight/obese, have a family history of diabetes, and have Asian ethnicity than women in the Non‐GDM group (*n* = 3562). They also had less gestational weight gain and more induced labors, while their infants were born earlier, had lower birthweight, and more neonatal hypoglycemia (all *p* < 0.001). Women in the Missed group had lower BMI and fewer were of Pacific ethnicity than the Both‐Criteria group (*n* = 151). They also had less pharmacotherapy use and postpartum hemorrhage.

**Conclusions:**

Using the revised criteria will increase GDM diagnoses by identifying a high‐risk group of women likely to benefit from treatment, but 35.5% of women currently treated for GDM will no longer be diagnosed, potentially compromising their health.

## Highlights

- Revising the gestational diabetes (GDM) diagnostic criteria in New Zealand will increase the prevalence of GDM by 1% and additionally identify a high‐risk group of women and their infants exposed to elevated fasting and 1‐h glucose concentrations on oral glucose tolerance tests, who will likely benefit from GDM treatment.
- As the revised criteria exclude a 2‐h glucose result, 35.5% of women currently receiving GDM care would no longer be diagnosed, potentially compromising their health and the health of their infants.

## Graphical abstract

Revising the New Zealand gestational diabetes (GDM) diagnostic criteria leads to an 18% relative increase of GDM prevalence by identifying an additional high‐risk group likely to benefit from treatment, but 35.5% of those currently receiving GDM care would no longer be diagnosed, potentially compromising their maternal and infant health.

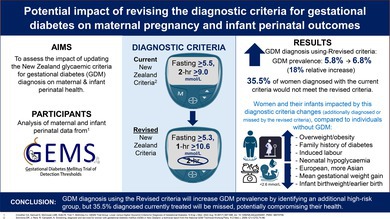

## 1 Introduction

Gestational diabetes mellitus (GDM) is a growing global health burden associated with significant maternal and child complications, yet there remains no international consensus on its optimal diagnostic criteria [1]. Various glycemic thresholds have been proposed for the fasting plasma glucose (FPG), 1‐h, 2‐h, and 3‐h post‐load glucose (PG) values on 75 or 100 g oral glucose tolerance test (OGTT) [2]. In New Zealand, the current GDM diagnostic criteria of a FPG ≥ 5.5 mmol/L and/or a 2‐h PG ≥ 9.0 mmol/L [3] were recommended by the New Zealand Society for the Study of Diabetes in 1991 [4]. A proposed change to these criteria recommends lowering the FPG to ≥ 5.3 mmol/L and replacing the 2‐h PG with a 1‐h PG ≥ 10.6 mmol/L. To date, no country has endorsed diagnosis of GDM based solely on FPG and/or 1‐h PG values, and evidence on the clinical impact of these changes is lacking. This study aimed to compare the demographics and perinatal health of women and their infants who (1) met only the revised criteria, (2) met only the current criteria, (3) met both criteria, or (4) met neither.

## 2 Methods

### 2.1 Study Design and Setting

This retrospective cohort study analyzed data from the GEMS (Gestational Diabetes Mellitus Diagnostic Detection Thresholds) randomized trial, conducted from April 2015 to August 2020 in Auckland, New Zealand. The trial enrolled women with a singleton pregnancy without pre‐existing diabetes or previous GDM [5]. Participants were randomized to GDM diagnosis using either the lower thresholds of the International Association of Diabetes in Pregnancy Study Groups (IADPSG) diagnostic criteria (FPG ≥ 5.1, 1‐h PLG ≥ 10.0 and/or 2‐h PLG ≥ 8.5 mmol/L) [6], or the higher thresholds of the current New Zealand criteria (FPG ≥ 5.5 and/or 2‐h PLG ≥ 9.0 mmol/L) [3]. Those diagnosed with GDM in the GEMS trial, according to their diagnostic criteria of randomization, received lifestyle advice and pharmacotherapy if needed for glycemic control. All participants provided written informed consent. Ethics approval was obtained from the Northern B Health and Disability Ethics Committee (13/NTB/18).

### 2.2 Definitions of Study Groups

For this study, participants were categorized into one of four groups based on their OGTT results at trial entry: (1) Additional group: met the revised criteria for GDM but not the current criteria (FPG 5.3 to 5.4, and/or 1‐h PG ≥ 10.6 mmol/L; 2‐h PG < 9.0 mmol/L), (2) Missed group: did not meet the revised criteria but met the current criteria (FPG < 5.3 and 1‐h PG < 10.6 mmol/L, but 2‐h PG ≥ 9.0 mmol/L), (3) Both‐Criteria group: met both criteria (either a) FPG ≥ 5.5 mmol/L, or (b) FPG 5.3 to 5.4 and/or 1‐h PG ≥ 10.6, and 2‐h PG ≥ 9.0 mmol/L, and (4) Non‐GDM group: met neither criteria (FPG < 5.3, 1‐h PG < 10.6 and 2‐h PG < 9.0 mmol/L).

All women in the Missed and Both‐Criteria groups had OGTT glucose concentrations exceeding the current New Zealand diagnostic criteria and therefore received treatment for GDM in the GEMS trial. However, among women in the Additional group, whose OGTT glucose concentrations fell between the higher New Zealand criteria and the lower IADPSG criteria, only women randomized to the lower IADPSG criteria group in GEMS received treatment for GDM (Additional‐Treated group), while those randomized to the higher New Zealand criteria group remained untreated (Additional‐Untreated group). Participants with FPG values of 5.1 to 5.2 mmol/L and/or 1‐h PG values of 10.0 to 10.5 mmol/L, who received treatment for GDM because they had been randomized to GDM diagnosis by the lower IADPSG criteria in the GEMS trial, were not eligible for inclusion in the current study, as they did not fit into any of our four study groups.

### 2.3 Study Outcomes

Maternal baseline characteristics at entry to the GEMS trial included maternal age, parity, body mass index (BMI) at first antenatal visit, overweight/obesity (BMI ≥ 25 kg/m^2^), self‐reported maternal ethnicity, and family history of diabetes. Maternal pregnancy‐related outcomes included preeclampsia, induction of labor, Cesarean section, postpartum hemorrhage (≥ 500 mL), gestational weight gain, pharmacotherapy for GDM, breastfeeding at hospital discharge, and length of postnatal stay. Infant outcomes included gestational age at birth, infant sex, birthweight, length and head circumference, and *z*‐scores [7], large for gestational age (LGA; birthweight > 90th percentile on standardized Fenton charts) [7], small for gestational age (SGA; birthweight < 10th percentile) [7], preterm (< 37 weeks' gestation), composite serious infant outcome (any of: perinatal death, death of liveborn before discharge, birth trauma, shoulder dystocia), neonatal hypoglycemia (glucose < 2.6 mmol/L requiring treatment), neonatal intensive care unit (NICU) admission, and length of postnatal stay.

### 2.4 Statistical Analysis

Baseline characteristics of participants in the Additional and Missed groups were compared with participants in the Both‐Criteria and the Non‐GDM groups. For maternal and infant health outcomes, participants in the Additional‐Treated and Missed groups were compared with participants in the Both‐Criteria and Non‐GDM groups. Subgroup analyses examined outcomes between the Additional‐Untreated, Additional‐Treated, and Non‐GDM groups.

Continuous variables are presented as mean ± standard deviation (SD); categorical variables as counts and proportions (%). Binary outcomes are reported as risk difference (RD) using identity‐link functions or relative risk (RR) using log‐link functions, and continuous outcomes as mean difference (MD) with identity‐link functions, all with 95% confidence intervals (CIs). To reduce type I error due to multiple comparisons, the significance level was adjusted using Bonferroni correction by dividing an initial alpha level (0.05) by four prespecified pairwise comparisons in each perinatal outcome analysis, yielding an adjusted significance level of *p* < 0.0125. All analyses were performed using SAS software version 9.4 (SAS Institute Inc., Cary, New Carolina, USA).

## 3 Results

### 3.1 GDM Prevalence and Characteristics in the Study Cohort

**FIGURE 1:**
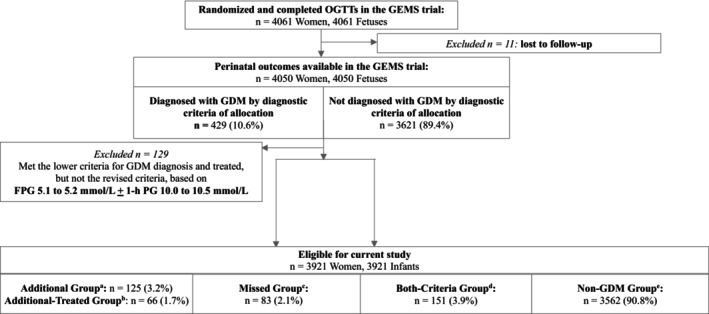
Flowchart of participants. ^a^Met only the revised criteria, did not meet the current criteria. ^b^Met only the revised criteria, did not meet the current criteria and received treatment for GDM. ^c^Met only the current criteria, did not meet revised criteria. ^d^Met both the current and revised criteria. ^e^Met neither the current nor the revised criteria.

**FIGURE 2:**
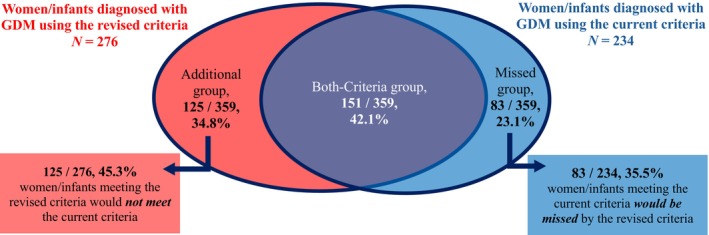
Venn diagram of 359 women/infants diagnosed with GDM in the current study, grouped by the revised and/or the current diagnostic criteria. 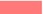 Additional group: Women/infants meeting the revised criteria but not the current criteria: FPG 5.3 to 5.4, and/or 1‐h PG ≥ 10.6 mmol/L; 2‐h PG < 9.0 mmol/L. 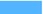 Missed group: Women/infants not meeting the revised criteria but met the current criteria: FPG < 5.3 and 1‐h PG < 10.6 mmol/L, but 2‐h PG ≥ 9.0 mmol/L. 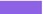 Both‐Criteria group: Women/infants meeting both criteria, either by FPG ≥ 5.5 mmol/L, or FPG 5.3 to 5.4 and/or 1‐h PG ≥ 10.6, and 2‐h PG ≥ 9.0 mmol/L. FPG, fasting plasma glucose; GDM, gestational diabetes mellitus; PG, post‐load glucose.

Among the 4050 mother–child pairs, the prevalence of GDM was 6.8% (276/4050, 95% CI 6.1% to 7.6%) using the revised criteria and 5.8% (234/4050, 95% CI 5.1% to 6.5%) using the current New Zealand criteria (risk ratio 1.18, 95% CI 1.00 to 1.40). Women who were not eligible for this study were slightly older than those who were more likely to be overweight or obese at trial entry, have a family history of diabetes, and less likely to be of Pacific ethnicity (Table 1). Among 3921 eligible mother–child pairs for the current study, 125 (3.2%) were in the Additional group, 83 (2.1%) in the Missed, 151 (3.9%) in the Both‐Criteria, and 3562 (90.8%) in the Non‐GDM group (Figure 1, Table 2). Thus, among the 359 of the 3921 participants who met either the current or the revised GDM criteria, 34.8% (125/359; 95% CI 30.1% to 39.9%) would be additionally diagnosed by only the revised criteria, while 23.1% (83/359; 95% CI 19.1% to 27.8%) currently diagnosed would not meet the revised criteria (RD 11.70%, 95% CI 5.12% to 18.28%; Figure 2). Overall, 45.3% of participants (125/276; 95% CI 39.5% to 51.1%) who met the revised criteria would have been missed by the current criteria, while 35.5% (83/234; 95% CI 29.6% to 41.9%) of participants meeting the current criteria would not be diagnosed by the revised criteria (Figure 2).

**TABLE 1:** Baseline characteristics in women included and excluded in this study.

	Included (*n* = 3921)	Excluded (*n* = 129)	*p* ^a^
Age, years (±SD)	31.4 ± 5.1	32.5 ± 5.2	0.023
Primiparity (%)	1896 (48.4%)	61 (47.3%)	0.811
BMI at first antenatal visit, kg/m^2^ (±SD)	27.4 ± 6.6	29.0 ± 5.8	0.087
Overweight/obesity (%)	2440 (61.2%)	98 (76.0%)	0.001
Family history of diabetes (%)	1352 (34.5%)	58 (45.0%)	0.018
Gestation at OGTT, weeks (±SD)	27.4 ± 1.6	27.6 ± 1.8	0.375
FPG, mmol/L (±SD)	4.4 ± 0.5	4.7 ± 0.4	< 0.001
1‐h PG, mmol/L (±SD)	7.4 ± 1.7	9.2 ± 1.2	< 0.001
2‐h PG, mmol/L (±SD)	6.1 ± 1.5	7.5 ± 1.2	< 0.001
Ethnicity (%)			
European	1580 (40.3%)	47 (36.4%)	0.370
Pacific	607 (15.5%)	12 (9.3%)	0.019
Māori	216 (5.5%)	7 (5.4%)	0.968
Asian	1286 (32.8%)	51 (39.5%)	0.123
Other	232 (5.9%)	12 (9.3%)	0.190

*Note:* Results are number (%) or mean (±SD).

Abbreviations: BMI, body mass index; FPG, fasting plasma glucose; OGTT, oral glucose tolerance test; PG, post‐load glucose; SD, standard deviation.

^a^ Statistical significance, defined as *p* < 0.05.

**TABLE 2:** Associations amongst baseline characteristics between the additional, missed, non‐GDM and both‐criteria groups.

	Additional group (*n* = 125)	Missed group (*n* = 83)	Non‐GDM group (*n* = 3562)	Both‐criteria group (*n* = 151)	RD/MD (95% CI), pairwise comparison *p* values^b^				Global *p* values^b^
					Additional vs. non‐GDM^a^	Additional vs. both‐criteria^a^	Missed vs. non‐GDM^a^	Missed vs. Both‐Criteria^a^	
Age, years (±SD)	32.0 ± 5.6	31.5 ± 4.4	31.4 ± 5.2	32.1 ± 4.7	0.63 (−0.28, 1.55), 0.176	−0.08 (−1.28, 1.15), 0.914	0.06 (−1.06, 1.17), 0.920	−0.64 (−2.02, 0.73), 0.360	0.225
Primiparity (%)	54 (43.2%)	50 (60.2%)	1716 (48.2%)	76 (50.3%)	−4.98 (−13.82, 3.87), 0.270	−7.13 (18.92, 4.66), 0.234	12.07 (1.41, 22.72), 0.027	9.91 (−3.30, 23.12), 0.142	0.089
BMI at first antenatal visit, kg/m^2^ (±SD)	30.6 ± 7.3	27.5 ± 5.5	27.8 ± 6.5	31.1 ± 7.9	2.87 (1.69, 4.05), < 0.001	−0.50 (−2.06, 1.06), 0.530	−0.22 (−1.66, 1.21), 0.760	−3.59 (−5.36, −1.83), < 0.001	< 0.001
Overweight/obesity (%)	97 (77.6%)	60 (72.3%)	2125 (59.7%)	118 (78.2%)	17.94 (10.46, 25.43), < 0.001	−0.55 (−10.39, 9.30), 0.914	12.63 (2.87, 22.40), 0.011	−5.86 (−17.53, 5.82), 0.325	< 0.001
Family history of diabetes (%)	56 (44.8%)	41 (49.4%)	1166 (32.7%)	89 (58.9%)	12.07 (3.21, 20.92), 0.008	−14.14 (−25.87, −2.41), 0.018	16.66 (5.79, 27.53), 0.003	−9.54 (−22.86, 3.78), 0.160	< 0.001
Gestation at OGTT, weeks (±SD)	27.5 ± 1.9	27.7 ± 1.7	27.4 ± 1.6	27.5 ± 1.7	0.05 (−0.25, 0.34), 0.754	−0.02 (−0.41, 0.37), 0.917	0.23 (−0.13, 0.58), 0.219	0.16 (−0.28, 0.60), 0.485	0.617
FPG, mmol/L (±SD)	5.0 ± 0.4	4.5 ± 0.4	4.3 ± 0.3	5.6 ± 0.9	0.65 (0.58, 0.72), < 0.001	−0.64 (−0.73, −0.55), < 0.001	0.15 (0.06, 0.23), < 0.001	−1.15 (−1.25, −1.04), < 0.001	< 0.001
1‐h PG, mmol/L (±SD)	10.3 ± 1.7	9.4 ± 0.9	7.1 ± 1.4	10.9 ± 1.8	3.11 (2.85, 3.36), < 0.001	−0.66 (−1.00, −0.33), 0.001	2.23 (1.92, 2.54), < 0.001	−1.54 (−1.92, −1.16), < 0.001	< 0.001
2‐h PG, mmol/L (±SD)	7.1 ± 1.2	9.7 ± 0.7	5.9 ± 1.2	9.4 ± 2.2	1.14 (0.91, 1.36), < 0.001	−2.37 (−2.66, −2.07), < 0.001	3.78 (3.51, 4.05), < 0.001	0.27 (−0.06, 0.61), 0.106	< 0.001
Ethnicity (%)									
European	21 (16.8%)	16 (19.3%)	1512 (42.5%)	31 (20.5%)	−25.65 (−32.40, −18.89), < 0.001	−3.73 (−12.92, 5.46), 0.426	−23.17 (−31.81, −14.53), < 0.001^b^	−1.25 (−11.91, 9.41), 0.818	< 0.001
Pacific	30 (24.0%)	7 (8.4%)	537 (15.1%)	33 (21.9%)	8.92 (1.34, 16.51), 0.021	2.15 (−7.83, 12.13), 0.673	−6.64 (−12.74, 0.55), 0.033	−13.42 (−22.32, −4.52), 0.003	0.003
Māori	12 (9.6%)	4 (4.8%)	194 (5.5%)	6 (4.0%)	4.15 (−1.07, 9.37), 0.119	5.63 (−0.41, 11.66), 0.068	−0.63 (−5.30, 4.04), 0.792	0.85 (−4.72, 6.41), 0.766	0.332
Asian	53 (42.4%)	50 (60.2%)	1111 (31.2%)	72 (47.7%)	11.21 (2.31, 20.01), 0.013	5.28 (−17.06, 6.49), 0.379	29.05 (18.41, 39.69), < 0.001	12.56 (−0.65, 25.77), 0.062	< 0.001
Other	9 (7.2%)	6 (7.2%)	208 (5.8%)	9 (6.0%)	1.36 (−3.24, 5.96), 0.562	1.24 (−4.66, 7.14), 0.680	1.39 (−4.24, 7.02), 0.628	1.27 (−5.46, 8.00), 0.712	0.906

*Note:* Results are number (%) or mean (±SD).

Abbreviations: BMI, body mass index; FPG, fasting plasma glucose; MD, mean difference; OGTT, oral glucose tolerance test; PG, post‐load glucose; RD, risk difference; SD, standard deviation.

^a^ The second groups in these pairwise comparisons are the reference groups.

^b^ Statistical significance following Bonferroni correction for prespecified pairwise comparisons, defined as *p* < 0.0125.

### 3.2 Baseline Demographics of the Additional and Missed Groups

Baseline characteristics of GEMS trial participants included and not included in the study are outlined in Table 1. Excluded women were slightly older, were more likely to be overweight or obese at trial entry, more likely to have a family history of diabetes, and less likely to be of Pacific ethnicity. Among participants included in this study, maternal age, parity, and gestation at OGTT were similar across the study groups, except for a slightly higher rate of primiparity in women in the Missed group compared with the Non‐GDM group (Table 2). Women in both the Additional and Missed groups, whose care would be altered by the change in criteria, had more overweight/obesity (77.6% and 72.3% vs. 59.7%; *p* < 0.001) than women in the Non‐GDM group, more family history of diabetes (44.8% and 49.4% vs. 32.7%; *p* < 0.001), fewer were European (16.8% and 19.3% vs. 42.5%; *p* < 0.001), and more were Asian (42.4% and 60.2% vs. 31.2%; *p* < 0.001). Women in the Additional group had a higher BMI at first antenatal visit than women in the Non‐GDM group (30.6 vs. 27.8 kg/m^2^; MD 2.87, 95% CI 1.69 to 4.05) and were more likely to be Pacific (24.0% vs. 15.1%; RD 8.92%, 95% CI 1.34% to 16.51%). Women in the Additional group, compared to women in the Both‐Criteria group, were less likely to have a family history of diabetes (44.8% vs. 58.9%; RD −14.14%, 95% CI −25.87% to −2.41%), whilst other characteristics were similar. Women in the Missed group, compared to those in the Non‐GDM and Both‐Criteria groups, were less likely to be Pacific (8.4% vs. 15.1% and 21.9%; *p* = 0.003), while their BMI was lower than women in the Both‐Criteria group (27.5 vs. 31.1 kg/m^2^; MD −3.59, 95% CI −5.36 to −1.83).

### 3.3 Maternal Pregnancy‐Related Outcomes

In the Additional group, 66 (52.8%) of participants received GDM treatment (Additional‐Treated), while 59 (47.2%) did not (Additional‐Untreated). Rates of pre‐eclampsia and Cesarean section were similar across study groups (Table 3). By hospital discharge, overall rates of breastfeeding were high and similar in all groups. Women in the Additional‐Treated and Missed groups, compared to those in the Non‐GDM group, had more induction of labor (57.6% and 50.6% vs. 29.0%; *p* < 0.001), lower mean gestational weight gain (9.5 and 9.7 vs. 11.8 kg; *p* < 0.001), and greater pharmacotherapy use for GDM treatment (80.3% and 61.5% vs. 0.4%; *p* < 0.001). Women in the Additional‐Treated group had generally comparable maternal outcomes to those in the Both‐Criteria group, except for less insulin use (36.4% vs. 52.3%; RD −15.95%, 95% CI −30.11% to −1.80%). Women in the Missed group appeared to have had longer mean postnatal stay than those in the Non‐GDM group (3.6 vs. 3.0 days; MD 0.60, 95% CI 0.15 to 1.04). Compared to those in the Both‐Criteria group, women in the Missed group had less postpartum hemorrhage and pharmacotherapy use, particularly insulin and combined therapy.

**TABLE 3:** Associations between maternal pregnancy‐related and infant perinatal outcomes between the Additional‐Treated, Missed, Non‐GDM and Both‐Criteria groups.

	Additional‐treated group (*n* = 66)	Missed group (*n* = 83)	Non‐GDM group (*n* = 3562)	Both‐criteria group (*n* = 151)	RR/MD (95% CI), pairwise comparison *p* values^f^				Global *p* values^f^
					Additional‐treated vs. non‐GDM^a^	Additional‐treated vs. both‐criteria^a^	Missed vs. non‐GDM^a^	Missed vs. both‐criteria^a^	
Maternal pregnancy‐related outcomes									
Preeclampsia (%)	0 (0.0%)	5 (6.0%)	130 (3.7%)	8 (5.3%)	NE, 0.975	NE, 0.974	1.65 (0.69, 3.93), 0.257	1.14 (0.38, 3.38), 0.817	0.516
Postpartum hemorrhage^b^, *n*/*N* (%)	19/65 (29.2%)	17/82 (20.7%)	1018/3498 (29.2%)	57/149 (38.3%)	1.00 (0.68, 1.47), 0.993	0.76 (0.50, 1.18), 0.221	0.71 (0.46, 1.09), 0.116	0.54 (0.34, 0.87), 0.011	0.028
Induction of labor (%)	38 (57.6%)	42 (50.6%)	1033 (29.0%)	85 (56.3%)	1.99 (1.60, 2.46), < 0.001	1.02 (0.80, 1.32), 0.860	1.74 (1.40, 2.17), < 0.001	0.90 (0.70, 1.16), 0.413	< 0.001
Cesarean section (%)	29 (43.9%)	28 (33.7%)	1295 (36.4%)	59 (39.1%)	1.21 (0.92, 1.59), 0.179	1.12 (0.80, 1.58), 0.496	0.93 (0.68, 1.26), 0.630	0.86 (0.60, 1.24), 0.426	0.473
Gestational weight gain, kg (±SD)	9.5 ± 5.2	9.7 ± 6.0	11.8 ± 6.9	9.0 ± 5.9	−2.29 (−3.99, −0.60), 0.008	0.47 (−1.54, 2.48), 0.649	−2.11 (−3.65, −0.58), 0.007	0.64 (−1.23, 2.52), 0.500	< 0.001
Breastfeeding at discharge (%)	66 (100.0%)	82 (98.8%)	3491 (98.0%)	146 (96.7%)	1.02 (NE), < 0.001	1.03 (NE), < 0.001^f^	1.01 (0.98, 1.03), 0.517	1.02 (0.98, 1.06), 0.266	0.537
Any pharmacotherapy for GDM^c^ (%)	53 (80.3%)	51 (61.5%)	15 (0.4%)	123 (81.5%)	79.88 (70.28, 89.48), < 0.001	−1.15 (−12.64, 10.33), 0.843	61.02 (50.55, 71.50), < 0.001	−20.01 (−32.24, −7.78), 0.001	< 0.001
Insulin use^c^ (%)	24 (36.4%)	19 (22.9%)	8 (0.2%)	79 (52.3%)	36.14 (24.53, 47.75), < 0.001	−15.95 (−30.11, −1.80), 0.027	22.67 (13.62 31.81), < 0.001	−29.43 (−41.54, −17.31), < 0.001	< 0.001
Oral hypoglycemic use^c^ (%)	43 (65.2%)	45 (54.2%)	12 (0.3%)	92 (60.9%)	64.81 (53.31, 76.32), < 0.001	4.22 (−9.74, 18.18), 0.552	53.88 (43.16, 64.60), < 0.001	−6.71 (−20.03, 6.61), 0.322	< 0.001
Insulin and oral hypoglycemic use^c^ (%)	14 (21.2%)	13 (15.7%)	5 (0.1%)	48 (31.8%)	21.07 (11.21, 30.94), < 0.001	−10.58 (−22.99, 1.84), 0.095	15.52 (7.70, 23.34), < 0.001	−16.13 (−26.97, −5.29), 0.004	< 0.001
Postnatal stay, days (±SD)	2.7 ± 1.5	3.6 ± 3.6	3.0 ± 2.0	3.1 ± 2.3	−0.24 (−0.74, 0.26), 0.342	−0.40 (−0.99, 0.19), 0.185	0.60 (0.15, 1.04), 0.008	0.44 (−0.11, 0.99), 0.114	0.033
Infant outcomes									
Gestational age at birth, weeks (±SD)	38.6 ± 1.2	38.3 ± 1.7	39.4 ± 1.6	38.1 ± 1.5	−0.83 (−1.21, −0.44), < 0.001	0.43 (−0.03, 0.89), 0.084	−1.11 (−1.45, −0.76), < 0.001	0.15 (−0.27, 0.58), 0.480	< 0.001
Preterm birth < 37 weeks’ gestation (%)	3 (4.6%)	7 (8.4%)	178 (5.0%)	19 (12.6%)	0.91 (0.30, 2.77), 0.868	0.36 (0.11, 1.19), 0.093	1.69 (0.82, 3.48), 0.156	0.67 (0.29, 1.53), 0.342	< 0.001
Infant female sex (%)	35 (53.0%)	36 (43.4%)	1664 (46.7%)	76 (50.3%)	6.26 (−5.89, 18.42), 0.313	2.36 (−12.10, 16.82), 0.749	−3.39 (−14.18, 7.40), 0.537	−7.29 (−20.63, 6.04), 0.284	0.515
Birthweight, g (±SD)	3195 ± 449	3094 ± 541	3416 ± 546	3213 ± 567	−221.53 (−354.40, −88.66), 0.001	−18.23 (−176.05, 139.60), 0.817	−322.50 (−441.26, −203.74), < 0.001	−119.19 (−265.35, 26.95), 0.119	< 0.001
Birthweight *z*‐score (±SD)	−0.1 ± 0.9	−0.2 ± 0.9	0.02 ± 0.9	0.1 ± 1.0	−0.12 (−0.35, 0.10), 0.286	−0.20 (−0.47, 0.07), 0.179	−0.26 (−0.46, −0.05), 0.014	−0.33 (−0.58, −0.08), 0.014	0.041
Birth length *z*‐score (±SD)	0.4 ± 1.0	0.1 ± 0.8	0.4 ± 1.0	0.4 ± 1.0	0.04 (−0.21, 0.29), 0.747	0.04 (−0.26, 0.33), 0.817	−0.26 (−0.49, −0.03), 0.024	−0.27 (−0.54, −0.0002), 0.050	0.154
Birth head circumference *z*‐score (±SD)	0.2 ± 1.0	0.1 ± 1.1	0.2 ± 1.1	0.3 ± 1.2	0.07 (−0.21, 0.34), 0.638	−0.10 (−0.43, 0.22), 0.559	−0.12 (−0.37, 0.13), 0.340	−0.29 (−0.60, 0.01), 0.078	0.224
LGA births^d^ (%)	4 (6.1%)	4 (4.8%)	313 (8.8%)	18 (11.9%)	0.69 (0.27, 1.79), 0.446	0.51 (0.18, 1.45), 0.206	0.55 (0.21, 1.44), 0.221	0.40 (0.14, 1.16), 0.092	0.263
SGA births^e^ (%)	8 (12.1%)	7 (8.4%)	284 (8.0%)	14 (9.3%)	1.52 (0.79, 2.94), 0.213	1.31 (0.57, 2.98), 0.522	1.06 (0.52, 2.17), 0.878	0.91 (0.38, 2.17), 0.831	0.605
Neonatal hypoglycemia requiring treatment (%)	10 (15.2%)	29 (34.9%)	258 (7.2%)	40 (26.5%)	2.09 (1.17, 3.74), 0.013	0.57 (0.30, 1.08), 0.084	4.82 (3.51, 6.61), < 0.001	1.32 (0.89, 1.96), 0.172	< 0.001
Composite serious infant outcome (%)	0 (0.0%)	1 (1.2%)	84 (2.4%)	6 (4.0%)	NE, 0.976	NE, 0.975	0.51 (0.07, 3.63), 0.502	0.30 (0.04, 2.50), 0.267	0.555
NICU admission (%)	3 (4.6%)	5 (6.0%)	142 (4.0%)	12 (8.0%)	1.14 (0.37, 3.48), 0.820	0.57 (0.17, 1.97), 0.375	1.51 (0.64, 3.59), 0.351	0.76 (0.28, 2.09), 0.591	0.096
Postnatal stay, days (±SD)	4.0 ± 3.9	5.3 ± 7.9	4.3 ± 6.1	4.9 ± 6.7	−0.27 (−1.77, 1.23), 0.725	−0.88 (−2.66, 0.90), 0.321	1.04 (−0.30, 2.38), 0.128	0.43 (−1.21, 2.08), 0.657	0.284

*Note:* Results are number (%) or mean (±SD).

Abbreviations: CI, confidence interval; gestational weight gain, gestational weight gain; LGA, large‐for‐gestational age; MD, mean difference; *N*, denominator, total no. of infants with available data for outcome analysis; *n*, numerator/total no. of infants with positive outcomes; NE, non‐estimable; NICU, neonatal intensive care unit; RD, risk differences; RR, relative risk; SD, standard deviation; SGA, small‐for‐gestational age.

^a^ The second groups in these pairwise comparisons are the reference groups.

^b^ 127 (3.2%) missing data.

^c^ Treatment effects are given as RD with 95% CIs.

^d^ LGA defined as birth weight above the 90th percentile for gestation and fetal sex on standardized birthweight charts (Fenton 2013) [7].

^e^ SGA defined as birth weight less than the 10th percentile for gestation and fetal sex on standardized birth weight charts by the population standards (Fenton 2013) [7].

^f^ Statistical significance following Bonferroni correction for prespecified pairwise comparisons, defined as *p* < 0.0125.

### 3.4 Infant Outcomes

Infants in both the Additional‐Treated and Missed groups, compared to those in the Non‐GDM group, had lower birthweight (3195 and 3094 vs. 3416 g; *p* < 0.001), were born earlier (38.6 and 38.3 vs. 39.4 weeks; *p* < 0.001), and had neonatal hypoglycemia detected and treated more often (15.2% and 34.9% vs. 7.2%; *p* < 0.001; Table 3). Infants in the Missed group had lower mean birthweight z‐scores than infants in the Non‐GDM and Both‐Criteria groups (−0.2 vs. 0.02 and 0.1; *p* = 0.041) and may have had a slightly lower birth length z‐score than infants in the Non‐GDM group (0.1 vs. 0.4; MD −0.33, 95% CI −0.58 to −0.08). Nevertheless, the rates of LGA and SGA births, preterm birth, infant sex, a composite serious neonatal outcome, NICU admission, and postnatal stay length were similar in all groups.

### 3.5 Subgroup Comparisons Between the Additional‐Untreated Versus Additional‐Treated and Non‐GDM Groups

Women in the Additional‐Untreated and Additional‐Treated groups were similar, except for fewer Asian women in the Additional‐Untreated group (Table 4). Women in the Additional‐Untreated group had lower rates of induced labor (39.0% vs. 57.6%; RR 0.68, 95% CI 0.46 to 0.99) and greater gestational weight gain (11.8 vs. 9.5 kg; MD 2.32, 95% CI 0.08 to 4.56) compared to women in the Additional‐Treated group. Women in the Additional‐Untreated group, compared to those in the Non‐GDM group, had higher BMI at first antenatal visit and higher rates of overweight/obesity, were less likely to be European, more likely to be Pacific, had higher rates of preeclampsia (10.2% vs. 3.7%; RR 2.79, 95% CI 1.28 to 6.06), but similar rates of Cesarean section, postpartum hemorrhage, and duration of postnatal stay. Infants in the Additional‐Untreated group, compared to infants in the Additional‐Treated and Non‐GDM groups, were more likely to be LGA (20.3% vs. 6.1% [*p* = 0.029] and 8.8% [*p* = 0.001]) with higher birthweight z‐score (0.4 vs. −0.1 [*p* = 0.005] and 0.02 [*p* = 0.002]), and were born earlier than infants in the Non‐GDM group (38.9 vs. 39.4 weeks; MD −0.47, 95% CI −0.88 to −0.06). Other neonatal complications were similar across groups.

**TABLE 4:** Subgroup analysis: associations between maternal pregnancy‐related and infant perinatal outcomes between the Additional group, who were untreated for GDM, versus those treated and those without GDM by either criteria.

	Additional‐untreated group (*n* = 59)	Additional‐treated group (*n* = 66)	Non‐GDM group (*n* = 3562)	RD/RR/MD (95% CI)			
				Additional‐untreated versus additional‐treated^a^	*p* ^f^	Additional‐untreated versus non‐GDM^a^	*p* ^f^
Baseline characteristics							
Age, years (±SD)	32.2 (6.2)	31.7 (5.0)	31.4 (5.2)	0.36 (−1.63, 2.34)	0.723	0.82 (−0.51, 2.15)	0.227
Primiparity (%)	23 (39.0%)	31 (47.0%)	1716 (48.2%)	1.07 (0.94, 1.20)	0.307	1.06 (0.96, 1.17)	0.235
BMI at first antenatal visit, kg/m^2^ (±SD)	31.1 (7.7)	30.3 (7.0)	27.8 (6.5)	0.78 (−1.81, 3.38)	0.552	3.28 (1.60, 4.97)	< 0.001
Overweight/obesity, (%)	48 (81.4%)	49 (74.2%)	2125 (59.7%)	0.93 (0.80, 1.08)	0.338	0.80 (0.73, 0.89)	< 0.001
Gestation at OGTT, weeks (±SD)	27.5 (1.8)	27.5 (1.9)	27.4 (1.6)	0.0044 (−0.65, 0.66)	0.990	0.05 (−0.37, 0.47)	0.819
FPG, mmol/L (±SD)	5.0 (0.5)	5.0 (0.4)	4.3 (0.3)	−0.034 (−0.19, 0.12)	0.668	0.63 (0.55, 0.72)	< 0.001
1−h PG, mmol/L (±SD)	10.3 (1.7)	10.2 (1.6)	7.1 (1.4)	0.14 (−0.45, 0.74)	0.635	3.18 (2.82, 3.55)	< 0.001
2−h PG, mmol/L (±SD)	7.0 (1.3)	7.1 (1.0)	5.9 (1.2)	−0.050 (−0.47, 0.37)	0.812	1.11 (0.80, 1.42)	< 0.001
Family history of diabetes (%)	24 (40.7%)	32 (48.5%)	1166 (32.7%)	0.92 (0.78, 1.10)	0.381	1.08 (0.95, 1.23)	0.218
Ethnicity (%)							
European	10 (17.0%)	11 (16.7%)	1512 (42.5%)	1.00 (0.88, 1.15)	0.966	0.77 (0.70, 0.85)	< 0.001
Pacific	18 (30.5%)	12 (18.2%)	537 (15.1%)	1.13 (0.97, 1.32)	0.110	1.17 (1.04, 1.31)	0.011
Māori	7 (11.9%)	5 (7.6%)	194 (5.5%)	1.04 (0.94, 1.16)	0.422	1.07 (0.98, 1.16)	0.129
Asian	19 (32.2%)	34 (51.5%)	1111 (31.2%)	0.82 (0.69, 0.98)	0.027	1.01 (0.90, 1.14)	0.869
Other	5 (8.5%)	4 (6.1%)	208 (5.8%)	1.02 (0.93, 1.12)	0.606	1.03 (0.96, 1.10)	0.470
Maternal pregnancy−related outcomes							
Preeclampsia (%)	6 (10.2%)	0 (0.0%)	130 (3.7%)	NE	NE	2.79 (1.28, 6.06)	0.010
Postpartum hemorrhage^b^, *n*/*N* (%)	18/58 (31.0%)	19/65 (29.2%)	1018/3498 (29.2%)	1.06 (0.62, 1.83)	0.828	1.06 (0.72, 1.57)	0.755
Induction of labor (%)	23 (39.0%)	38 (57.6%)	1033 (29.0%)	0.68 (0.46, 0.99)	0.047	1.34 (0.97, 1.86)	0.073
Cesarean section (%)	23 (39.0%)	29 (43.9%)	1295 (36.4%)	0.89 (0.58, 1.36)	0.577	1.07 (0.78, 1.48)	0.671
Gestational weight gain, kg (±SD)	11.8 (6.8)	9.5 (5.2)	11.8 (6.9)	2.32 (0.08, 4.56)	0.043	0.03 (−1.88, 1.93)	0.979
Any pharmacotherapy for GDM^c^ (%)	2 (3.4%)	53 (80.3%)	15 (0.4%)	−76.91 (−87.67, −66.16)	< 0.001	2.97 (−1.66, 7.59)	0.208
Postnatal stay, days (±SD)	2.8 (1.9)	2.7 (1.5)	3.0 (2.0)	0.07 (−0.52, 0.66)	0.818	−0.17 (−0.68, 0.34)	0.511
Infant perinatal outcomes							
Gestational age at birth, weeks (±SD)	38.9 (1.2)	38.6 (1.2)	39.4 (1.6)	0.35 (−0.07, 0.78)	0.102	−0.47 (−0.88, −0.06)	0.023
Preterm birth < 37 weeks gestation (%)	2 (3.4%)	3 (4.6%)	178 (5.0%)	0.75 (0.13, 4.39)	0.744	0.68 (0.17, 2.67)	0.579
Female infant (%)	32 (54.2%)	67 (53.6%)	1664 (46.8%)	1.01 (0.85, 1.21)	0.893	1.08 (0.95, 1.23)	0.254
Birthweight *z*‐score (±SD)	0.4 (1.0)	−0.1 (0.9)	0.02 (0.9)	0.50 (0.16, 0.85)	0.005	0.38 (0.14, 0.62)	0.002
Birth length *z*‐score (±SD)	0.5 (1.1)	0.4 (1.0)	0.4 (1.0)	1.05 (0.72, 1.54)	0.786	0.09 (−0.17, 0.36)	0.486
Birth head circumference z‐score (±SD)	0.3 (1.1)	0.2 (1.0)	0.2 (1.1)	0.09 (−0.29, 0.47)	0.641	0.16 (−0.13, 0.44)	0.290
LGA births^d^ (%)	12 (20.3%)	4 (6.1%)	313 (8.8%)	3.36 (1.13, 9.95)	0.029	2.31 (1.38, 3.88)	0.001
SGA births^e^ (%)	1 (1.7%)	8 (12.1%)	284 (8.0%)	0.14 (0.02, 1.11)	0.062	0.21 (0.03, 1.49)	0.119
Composite serious infant outcome (%)	3 (5.1%)	0 (0.0%)	84 (2.4%)	NE	NE	2.16 (0.70, 6.63)	0.180
Neonatal hypoglycemia needing treatment (%)	5 (8.5%)	10 (15.2%)	258 (7.2%)	0.56 (0.20, 1.56)	0.264	1.17 (0.50, 2.73)	0.718
NICU admission (%)	0 (0.0%)	3 (4.6%)	142 (4.0%)	NE	NE	NE	NE
Length of postnatal stay, days (±SD)	3.4 (2.0)	4.0 (3.9)	4.3 (6.1)	−0.64 (−1.74, 0.46)	0.252	−0.91 (−2.47, 0.65)	0.254

*Note:* Results are number (%) or mean (±SD).

Abbreviations: BMI, body mass index; CI, confidence intervals; FPG, fasting plasma glucose; GDM, gestational diabetes; gestational weight gain, gestational weight gain; LGA, large‐for‐gestational age; MD, mean difference; *N*, denominator/total no. of infants with available data for outcome analysis; *n*, numerator/total no. of infants with positive outcomes; NE, non‐estimable; NICU, neonatal intensive care unit; OGTT, oral glucose tolerance test; PG, post‐load glucose; RD, risk difference; RR, relative risk; SD, standard deviation; SGA, small‐for‐gestational age.

^a^ The second groups in these pairwise comparisons are the reference groups.

^b^ 66 (1.8%) missing data.

^c^ Treatment effects are given as RD with 95% CIs.

^d^ LGA defined as birth weight above the 90th percentile for gestation and fetal sex on standardized birthweight charts (Fenton 2013) [7].

^e^ SGA defined as birth weight less than the 10th percentile for gestation and fetal sex on standardized birth weight charts by the population standards (Fenton 2013) [7].

^f^ Statistical significance defined as *p* < 0.05.

## 4 Discussion

In this retrospective cohort analysis, we examined the potential impact of adopting the revised GDM criteria in New Zealand on GDM prevalence, demographic characteristics, and perinatal health. We found the prevalence of GDM would increase from 5.8% to 6.8% with use of the revised criteria, rates which are in keeping with national data [8] and consistent with prior literature reporting lower glycemic thresholds are associated with an increased prevalence of GDM being diagnosed [9]. Despite the small sizes of the Additional and Missed groups, the proposed diagnostic criteria change would result in a relative 18.0% and 11.7% absolute increase in total GDM caseload. Nearly half of the women diagnosed with GDM by the revised criteria would not be identified with the current criteria, while over one‐third of those currently diagnosed with GDM would no longer be diagnosed and treated. Overall, these changes will increase demand for healthcare resources but exclude high‐risk individuals who are currently benefiting from care.

Our finding of more non‐European women in the Additional and Missed groups is consistent with reports of higher GDM prevalence among ethnic minorities in New Zealand [10, 11]. Māori representation was similar across all groups, suggesting no additional impact of the revised criteria on GDM diagnoses for women of Māori ethnicity. However, unlike earlier studies suggesting Māori women are more often diagnosed based on FPG values ≥ 5.1 mmol/L [12, 13], we found no increased representation of Māori ethnicity in the Additional group, despite the revised FPG threshold being lowered from ≥ 5.5 to ≥ 5.3 mmol/L. In contrast, we observed more Pacific women in the Additional group. Pacific women are more commonly diagnosed with GDM based on an elevated FPG compared with women of European and South‐East Asian ethnicities [12, 14]. This is often associated with higher maternal BMI and greater need for insulin therapy that may predispose to FPG abnormalities, which would be identified using the lower revised FPG threshold [14, 15]. The higher proportion of Asian women in the Missed group is consistent with large, multi‐ethnic cohort studies reporting that Asian women are more likely to have GDM diagnosed by an elevated post‐load glucose despite normal FPGs compared with other ethnicities [12, 16, 17]. The post‐load hyperglycemia phenotype observed in Asian women likely reflects a combination of lower beta‐cell insulin secretory capacity [18], relative differences in body fat distribution with reduced muscle mass [12] and genetic predisposition [19].

Because all women in the Missed and Both‐Criteria groups, but only a subset of women in the Additional group, received treatment for GDM—our primary analyses of perinatal outcomes were restricted to comparisons involving only participants in the Additional‐Treated group, other treated participants, and those without GDM. This approach aimed to isolate the effects of differences in diagnostic thresholds rather than treatment effects in comparisons between the three treated groups.

Women in the Additional‐Treated group and the Missed group had higher rates of overweight/obesity and family history of diabetes, but less gestational weight gain and no increase in most pregnancy and neonatal morbidities compared to those in the Non‐GDM group. In contrast, those in the Additional‐Untreated group had higher rates of pre‐pregnancy overweight/obesity, preeclampsia, and LGA births compared to women in the Non‐GDM group. These findings suggest that women in both the Additional and Missed groups represent metabolically higher‐risk populations than those in the Non‐GDM group and would or may have benefitted from GDM treatment. These findings support previous randomized trials reporting benefits of treating mild GDM [20, 21] diagnosed using several different diagnostic criteria.

The largely similar outcomes between treated participants in the Additional and Both‐Criteria groups, along with worse outcomes in the Additional‐Untreated group, support treatment for individuals who will be additionally diagnosed with GDM by the revised criteria. These findings are consistent with a prospective observational study reporting GDM diagnosis using the higher National Institute for Health and Care Excellence criteria [22], compared to the lower Canadian criteria [23] and IADPSG criteria [6], have failed identifying women with increased biomarkers of insulin resistance, who may have benefitted from GDM treatment [24].

A diagnosis of GDM increased the use of induction of labor, likely as a result of clinician awareness of the clinical practice guideline recommendation [3] on induction for women with GDM [25]. Induction of labor may have contributed, in turn, to the earlier gestational age at birth and lower birthweight of infants in both the Additional‐Treated and Missed groups compared to those unexposed to GDM, which potentially mitigated against serious infant outcomes [26]. The higher rates of treated neonatal hypoglycemia in both the Additional‐Treated and Missed groups likely reflect the recommendation to test for hypoglycemia and therefore increased testing in infants exposed to GDM [27]. The rates of SGA were reassuringly similar across all groups in our study.

Women in the Missed group had a lower BMI at their first antenatal visit compared to women in the Both‐Criteria group. This likely resulted from the lower proportion of Pacific women in the Missed group, among whom higher BMI is more common [10, 12, 28]. The lower postpartum hemorrhage rate in women in the Missed group may also be a result of their lower BMI than those in the Both‐Criteria group, as high BMI is a known risk factor for postpartum hemorrhage [29]. The reduced likelihood of pharmacotherapy use for the treatment of GDM in women in the Missed group, compared to those meeting both criteria, particularly insulin, is consistent with a recent systematic review that reported women diagnosed with GDM based only on an elevated 2‐h PG were less likely to have required insulin for treating their GDM. This may reflect better treatment response in women in the Missed group, with their primary pathophysiology being insulin resistance without increased hepatic glucose production, as indicated by their normal FPG and 1‐h PG values [30].

Removing the 2‐h PG value for GDM diagnosis in the revised criteria reflects both stakeholder feedback regarding the practical challenges of performing a 2‐h OGTT [31], and the findings in the Hyperglycemia and Adverse Pregnancy Outcome study that FPG and 1‐h PG values better predict adverse perinatal outcomes than a 2‐h PG [32]. Our results suggest the revised criteria would exclude high‐risk women in the Missed group from blood glucose surveillance during pregnancy, in addition to lifestyle interventions, and pharmacotherapy for gestational hyperglycemia. As shown in previous randomized trials, untreated women in the Missed group are likely to have higher risks for preeclampsia, a LGA infant, and serious perinatal complications including shoulder dystocia [20, 21], although their rate of induced labor may be lower than in individuals with treated GDM. Failure to diagnose and treat GDM in this high‐risk group may thus compromise maternal and infant health.

Strengths of this study include our large, well‐characterized and contemporary cohort using data from the GEMS randomized trial [5], which enhances the relevance and reliability of our findings to the evolving New Zealand GDM diagnostic criteria. A key limitation is that all women in the Missed group received treatment for their GDM. Consequently, we are unable to assess how the revised criteria would have affected this group, who would no longer have been diagnosed or treated for GDM. Without treatment, uncontrolled maternal hyperglycemia during pregnancy can increase fetal glucose exposure and in utero hyperinsulinemia [33, 34], which are well‐established risk factors for adverse maternal and neonatal outcomes [20, 21, 34]. An additional limitation is the small sample size of this study, which may have restricted the ability to detect small but potentially clinically important between‐group differences and reduced statistical precision. For this reason, results are interpreted with the reported effect measures and CIs rather than relying on *p* values.

In conclusion, adopting the revised GDM diagnostic criteria will increase the prevalence of GDM, identify more high‐risk women who may benefit from treatment, while excluding a slightly smaller yet similarly high‐risk group currently diagnosed with GDM and benefiting from treatment. During the transition to adoption of the revised diagnostic criteria, consideration could be given to measuring both the 1‐h and 2‐h PG values in women undergoing OGTTs and assessing the maternal and infant outcomes initially using the current criteria, and then moving to the revised criteria. Future research should also evaluate the long‐term impact of the proposed changes in glycemic criteria for GDM diagnosis.

## Funding

This study was funded by a grant from the Health Research Council of New Zealand (HRC), grant 19/690, and a clinical research training fellowship from the HRC, ref. 25/002.

## Conflicts of Interest

The authors declare no conflicts of interest.

## Data Availability

Research data are not shared.
